# Validity of Short-Term Assessment of Risk and Treatability in the Japanese Forensic Probation Service

**DOI:** 10.3389/fpsyt.2021.645927

**Published:** 2021-05-05

**Authors:** Akiko Kikuchi, Takahiro Soshi, Toshiaki Kono, Mayuko Koyama, Chiyo Fujii

**Affiliations:** Department of Community Mental Health and Law, National Institute of Mental Health, National Center of Neurology and Psychiatry, Tokyo, Japan

**Keywords:** short-term assessment of risk and treatability (START), risk assessment, predictive validity, strength, structured professional judgement, forensic, outpatient

## Abstract

This study aimed to evaluate the predictive validity and reliability of the Short-Term Assessment of Risk and Treatability (START) in the context of the Japanese forensic probation service. START is a structured professional judgement guide for risk domains concerning negative behaviors such as violence, self-harm, suicide, substance abuse, unauthorized leave, victimization, and self-neglect. In this study, rehabilitation coordinators evaluated community-dwelling patients who were treated under the Medical Treatment and Supervision Act at baseline and followed-up for 6 months. The results revealed that START vulnerability scores significantly predicted self-harm, suicide, physical aggression, substance abuse, and self-neglect. START strength scores predicted physical violence and unauthorized leave. Specific risk estimates predicted physical violence and self-neglect. Risk judgement for future substance use may require adjustments for cultural differences, because of the lower prevalence in Japan. These results suggest that START offers a feasible and valid tool that allows clinicians to plan treatment and promote recovery of forensic patients in Japan.

## Introduction

Violence is not the only negative behavior in the prognosis of Mentally Disordered Offenders (MDOs). Suicide, self-harm, substance abuse, self-neglect, and victimization have all been found to occur at higher rates among psychiatric patients than among the general population (1–4). Risk management of these problem behaviors is a routine practice in psychiatry. Logically, risk management of such behaviors requires risk assessment tools for as many problem behaviors as there are. However, risk factors underlying different problem behaviors are known to overlap (5). A tool that can aggregate assessment items and assess the risk of worrisome outcomes for each patient would therefore be desirable.

The purpose of risk assessment in clinical practice is to guide treatment planning to support the recovery of the individual. One important perspective to support recovery in mental health is the focus on strength (6, 7). Focusing on strength has been shown to not only restore self-esteem and improve quality of life in subjects, but also improve social functioning and reduce risk behaviors (6). Strength is a protective factor that mitigates risk (8).

In the past decade, the interest in incorporating protective factors into the risk assessment and management of violence has been growing. Relevant clinical research has developed various risk measures that include protective factors, such as the Short-Term Assessment of Risk and Treatability (START) (8, 9), Structured Assessment of Violence Risk in Youth (SAVRY) (10), and Structured Assessment of Protective Factors for Violence Risk (SAPROF) (11).

The START is a risk assessment guide for a series of negative outcomes in psychiatric patients, such as violence, self-harm, suicide, self-neglect, victimization, substance abuse, and unauthorized leave. *This guide was developed as a short-term risk assessment for people with mental illness, substance use, and personality disorders*. Unlike traditional vulnerability-focused approaches, START assesses empirically selected dynamic factors comprising both protective factors (“strengths”) and risk factors (“vulnerabilities”), and judges the risk of seven negative outcomes occurring over a pre-determined period, as specific risk estimates (SREs). The negative outcomes of interest are: violence, self-harm, suicide, substance use, unauthorized leave, victimization, and self-neglect.

According to a systematic review of START, vulnerability score, strength score, and SREs have all been shown to have predictive validity for violent outcomes (12). With regard to outcomes other than violence, a meta-analysis suggested that although neither vulnerability nor strength scores predicted self-harm, the SRE for self-harm did offer predictive validity (12). Only one study has reported adequate predictive validities for unauthorized leave and substance use, although predictive validities for self-neglect and victimization were not significant (13).

Results from these previous studies were promising, but further evaluation may be necessary for at least two reasons. First, past studies of START were conducted in Western countries [e.g., Canada, Norway, Australia, the United Kingdom (UK), the United States, the Netherlands]. Cross-cultural generalization of START may be crucial to clarify whether the same risk and strength factors can predict negative outcomes in different cultures, such as Asian samples.

Second, most studies have examined the predictive validity of START in inpatient populations. For example, Nicholls et al. reported that case managers used START to assess patients in the community, but did not investigate its predictive validity (14). Another study tested the psychometric characteristics of START for 301 outpatient forensic psychiatric patients in the Netherlands (15). They found that for the 6-month follow-up, structured professional judgement ratings by the clinicians modestly improved the prediction of future violence beyond a summation of historical, vulnerability, and strength scores. To the best of our knowledge, no other studies have investigated the predictive validity of START in forensic outpatients.

In summary, research to date on the START has focused mainly on inpatients in Western countries. As START adopts a Structured Professional Judgement (SPJ) approach (16), the items were selected by a comprehensive review of the literature on risk factors for negative outcomes in psychiatric patients (8). Unlike actuarial risk assessments, item selection did not rely on a specific sample on which the assessment was developed. We thus expect that the predictive accuracy may be generalizable to other samples, such as Asian countries. To expand the literature on START, this study provides a first examination of the predictive validity of START in a Japanese forensic outpatient context.

## Materials and Methods

### Study Design

This study comprised a 6-month prospective study of outpatients in the community in Japan under the “Act for the Medical Treatment and Supervision of Persons with Mental Disorders Who Caused Serious Harm,” commonly called the “Medical Treatment and Supervision Act (MTSA).” The follow-up period of 6 months was selected for two reasons. First, a short term was required, as START was deliberately developed to assess short-term risk and treatability. Second, the period of follow-up had to be long enough for incidents to occur, as a past study showed that the rate of reoffending within 1 year after discharge was <3% among MTSA patients (17), and cosiderably low rate. Therefore, it was assumed better to follow up for 6 months rather than three, in order to increase the chance of collecting negative incident data.

### Setting

The MTSA in Japan is a forensic mental health act for Mentally Disordered Offenders (MDOs) who have committed murder, severe injury, arson, robbery, rape, or indecent assault under a state of insanity or diminished criminal responsibility. The act was passed by the parliament in 2003 and came into effect in 2005. When MDOs are introduced to the MTSA system and are mandated by the district court as warranting treatment under the MTSA, they are allocated to receive either an inpatient treatment order or an outpatient treatment order (18). The MTSA stipulates that the outpatient treatment order can last up to 3 years and be prolonged up to 5 years in total under special circumstances, but no longer. Past studies have found that the total cumulative rate of reoffending after discharge was 2.5% (1.1–3.9%) at 1 year and 7.5% (4.6–10.4%) at 3 years. The rate of serious reoffending was 0.4% (0.18–0.99%) at 1 year and 2.0% (0.4–3.6%) at 3 years (17).

### Participants

#### Inclusion Criteria

Patients were included when they had been given an MTSA outpatient treatment order by the district court and were dwelling in the community.

#### Exclusion Criteria

Exclusion criteria were as follows:

1. If the outpatient treatment order was known to expire within 6 months. The maximum MTSA outpatient treatment order is 5 years. Therefore, for example, if a patients' outpatient treatment had exceeded 4.5 years, it was apparent that the outpatient treatment order would expire before 6 months.
2. When the patient was under an MTSA outpatient treatment order, but was hospitalized in a psychiatric unit under the Mental Health and Welfare Act at Time 1. The MTSA stipulates that patients can be hospitalized under the Mental Health and Welfare Act for regular psychiatric care while remaining under the MTSA outpatient treatment order. Such patients were excluded from this study as their situation could not be considered to represent “living in the community.”

### Procedure

The START manual was translated into Japanese by the authors with formal written consent from the original authors. The first author had experience in the SPJ scheme and participated in a START workshop by the original authors prior to the beginning of the study.

Rehabilitation coordinators (RCs) were recruited in collaboration with the Mental Health Probation Planning Office in the Ministry of Justice. RCs are forensic probation officers who provide supervision and case management of MTSA patients. RCs regularly meet with MTSA patients, and hold care coordination meetings with the participation of related caregivers and agencies in the community. RCs gather information about the patient to monitor, supervise, and coordinate treatment efforts. RCs are responsible for collating any incident reports.

Those RCs who provided informed consent to participate in the study were provided with the Japanese START manual and received 1 day of training in scoring START. All training was provided by a clinical psychologist (first author). Training was conducted in eight regions regulated by the Regional Branch Bureau of Health and Welfare in Japan. After training, RCs were able to contact the first author for clarifications pertaining to the scoring of items in START. Only two of 102 RCs were trained in the use of any SPJ instrument prior to this study.

After START training, data collection was longitudinally implemented in two parts. At Time 1, RCs were asked to score the START of patients in their caseload who met the inclusion criteria for the study. RCs were required to use the START and estimate the risks of the seven negative outcomes during the 6 months subsequent to the assessment. Completed START summary sheets were then sent to the Mental Health Probation Planning Office. RCs were asked to maintain records of challenging behaviors from patients for the next 6 months as in routine practice. This information was to be posted to the problematic behavior form. At Time 2, 6 months after Time 1, RCs sent the problematic behavior forms to the Mental Health Probation Planning Office. All data sheets were anonymized in the Mental Health Probation Planning Office before being sent to the first author for analyses.

### Measures

For each eligible patient, RCs completed START, and a sociodemographic face-sheet at Time 1, and the problematic behaviors form designed specifically for this study at Time 2.

#### Short-Term Assessment of Risk and Treatability (START)

Unlike traditional vulnerability-focused approaches, START assesses 20 empirically selected dynamic factors (Table 1) in terms of both protective factors (strengths) and risk factors (vulnerabilities). Raters can add up to two case-specific items. Protective and risk factors are rated independently on three levels: 0 = minimal or no vulnerability/strength; 1 = moderate vulnerability/strength; and 2 = high vulnerability/strength. The evaluator also identifies critical vulnerabilities and key strengths, signature risk signs, medical conditions, and histories of the seven negative outcomes. Finally, the evaluator rates the risk of each outcome occurring over a predetermined period on a scale of low, medium, and high. A rating of low risk indicates no or minimal risk, moderate indicates greater than average risk, and high indicates a relatively imminent and serious threat.

**Table 1:** Items of the Short-Term Assessment of Risk and Treatability (START).

1.	Social skills
2.	Relationships
3.	Occupational
4.	Recreational
5.	Self-care
6.	Mental state
7.	Emotional state
8.	Substance use
9.	Impulse control
10.	External triggers
11.	Social support
12.	Material resources
13.	Attitudes
14.	Medication adherence
15.	Rule adherence
16.	Conduct
17.	Insight
18.	Plans
19.	Coping
20.	Treatability
21. and 22.	Case specific items

START has shown practical utility when incorporated into routine practice. Nicholls et al. (19) found excellent inter-rater reliability overall (intraclass correlation coefficient, ICC2 = 0.87, *p* < 0.001). Doyle et al. assessed START implementation, recruiting staff members of a medium secure forensic mental health service who had participated in the START training (20). They found that START took a mean of 25 min to complete, and 82.1% of assessments were completed in ≤30 min. Another study conducted in a UK medium secure hospital found that, by the second application of START, professionals were able to complete the assessment in 11.03 min (21). START was identified as a tool supporting best practice in managing violence as well as related risks among psychiatric patients in the UK (22).

This study excluded case-specific items from the analysis, because these were specific to individuals and not comparable between patients. Total scores on strength and vulnerability items were prorated to account for up to four missing items in accordance with the START manual (8). According to the recommendation in the START manual, assessments with more than five or more missing item data were excluded (8).

#### Problematic Behavior Data

Outcome measures were problematic behaviors exhibited by the patient. Data collection was operationalized by asking RCs to write down the problematic behaviors and then to categorize each event into one of the following: self-harm, suicide, physical violence, substance abuse, victimization, self-neglect, unauthorized leave, or other challenging behaviors (free description). Data for other challenging behaviors (e.g., water intoxication) were not included in this study.

#### Demographic and Clinical Data

Data on age, sex, diagnosis (International Classification of Diseases, 10th edition (ICD-10) (23), index offense, and length of MTSA outpatient treatment were collected at Time 1. The information in patient records were transferred into the dataset. The diagnoses were decided by certified psychiatrists who implemented the court-ordered mental health examination for 3 months. The ICD-10 system is used for MTSA diagnoses. The mental health examination report was submitted to the district court to be reviewed in the process of making decisions about the case. In rare instances where the main diagnosis is proven to be different during the MTSA treatment, the diagnosis is renewed accordingly in the official patient records.

### Analyses

Receiver operating characteristic (ROC) analyses were used to examine the predictive validity of START Vulnerability and Strength scores, and SREs for the different challenging behavior incidents in the 6 months following the Time 1 evaluation. ROC analysis has been widely used in violence prediction research due to its independence from base rates (24). To quantify the ROC, area under the ROC curve (AUC) was calculated. Strength scores were inverted when conducting ROC analyses to compare predictive validity to the total vulnerability score and specific risk estimates. Spearman's rho between the START vulnerability score, strength score, and the number and type of problematic challenging behaviors was calculated. Cronbach's alpha was used to measure the internal consistency of START items. All analyses were conducted using SPSS version 21.0 software (IBM corporation, Armonk, NY).

## Results

### Study Profile

**Figure 1:**
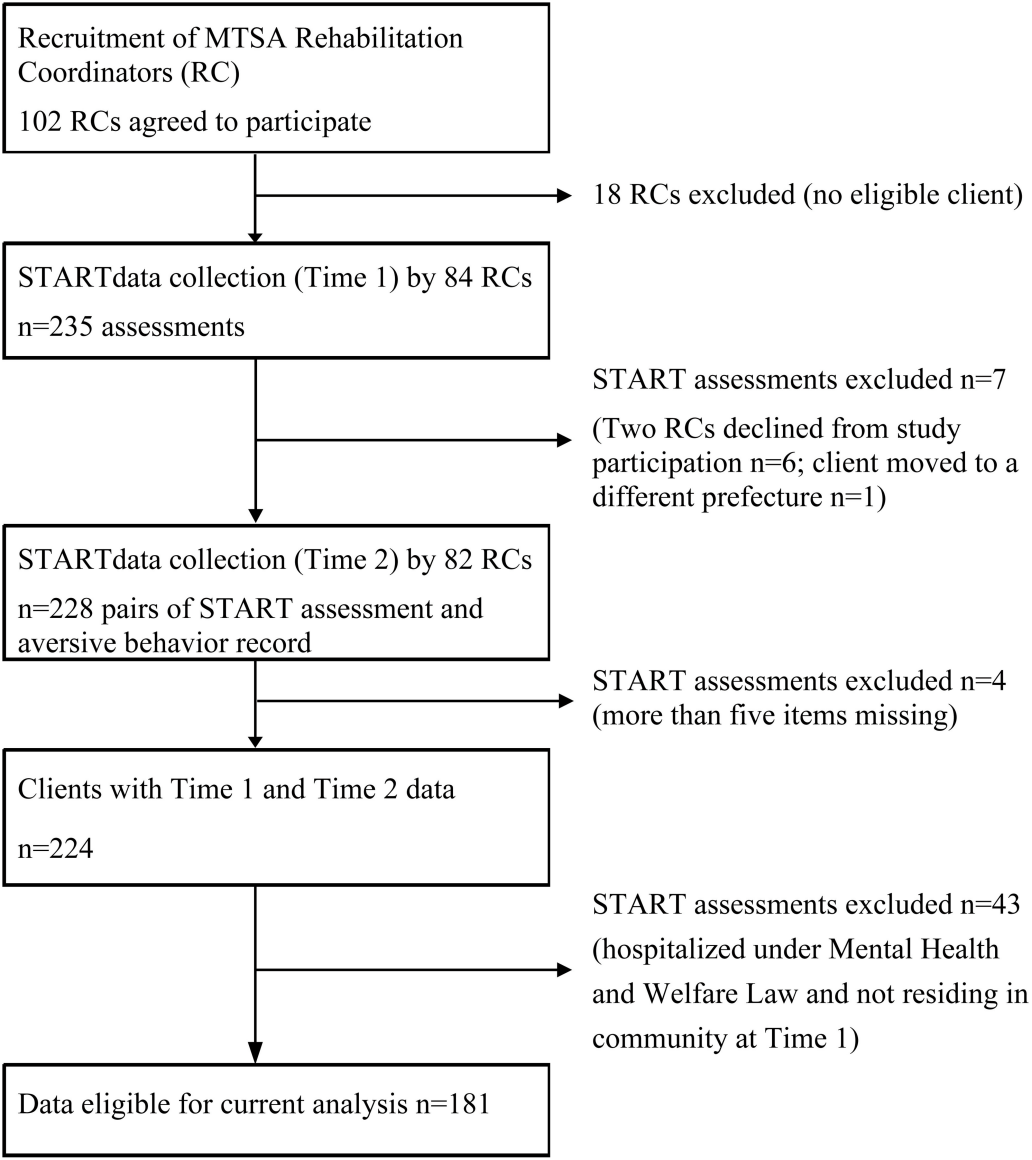
Flow diagram of data collection.

In total, 102 RCs (57.6% of the total number of RCs in Japan at Time 1) were recruited to the study, of whom 18 were excluded owing to an absence of eligible patients in their caseload (Figure 1). At Time 1, a total of 235 START assessments were completed by 84 RCs. By Time 2, 6 months after initial assessment, two RCs declined to participate in the study, resulting in a decrease of six START assessments. Another START assessment was excluded due to a patient moving to another prefecture. As a result, 228 pairs of START assessment and problematic behavior forms were obtained. Based on the exclusion criterion of START assessments with more than five missing item scores, four patients were further excluded from the analysis based on recommendations in the START manual (8). Another 43 assessments were excluded as the client was hospitalized under the Mental Health and Welfare Act at Time 1. As a result, 181 pairs of START assessments from Time 1 and problematic behavior forms from Time 2, were analyzed in this study.

#### Sample Characteristics

Table 2 shows descriptive characteristics of the study. The 181 eligible study subjects comprised predominantly men (79%) with a mean age of 43 years (range 24–86 years). The most frequent ICD-10 diagnosis was F2, schizophrenia (*n* = 141; 77.9%). The second most frequent was F1 (*n* = 19; 10.5%), Mental and Behavioral Disorders due to Psychoactive Substance Use. Concerning the index offense, murder, injury, and arson made up to ~90% of the total number. At Time 1, the average length of MTSA outpatient treatment was 14.46 months [standard deviation (SD) = 8.66 months]. Overall, the study sample did not significantly differ from the national MTSA sample in terms of distributions of gender, age at Time 1, diagnosis, or index offense (25).

**Table 2:** Descriptive statistics at Time 1 (*n* = 181).

		**n/Mean**	**%/SD**
Gender	Male	143	79.00
	Female	38	21.00
Age (years)		42.75	12.51
Diagnosis (ICD-10)			
	F0	2	1.10
	F1	19	10.50
	F2	141	77.90
	F3	9	4.97
	F4	3	1.66
	F6	1	0.55
	F7	2	1.10
	F8	2	1.10
	G4	2	1.10
Index offense			
	Murder	58	32.0
	Injury	64	35.4
	Arson	42	23.2
	Robbery	7	3.9
	Rape	3	1.7
	Indecent assault	7	3.9
MTSA outpatient treatment (months)		14.46	8.66

*F0, organic and symptomatic mental disorders; F1, mental and behavioral disorders due to psychoactive and other substance use; F2, schizophrenia, schizotypal, and delusional disorders; F3, mood or affective disorders; F4, neurotic, stress-related, and somatoform disorders; F6, disorders of adult personality and behavior; F7, mental retardation; F8, disorders of psychological development; G4, epilepsy and recurrent seizures*.

#### START Scores

In 6 months, 42 patients (23.2%) showed at least one START negative outcome (Table 3). The most commonly observed negative outcome was self-neglect, in 24 patients (13.2%). The least common risk outcomes were self-harm and victimization [two patients (1.10%) each]. No participants were rated as high risk for victimization or unauthorized leave. Mean vulnerability score was 12.52 (SD = 7.40) and mean strength score was 23.55 (SD = 7.90). Vulnerability score correlated negatively with strength score (Spearman's rho = −0.55, *p* < 0.01).

**Table 3:** Distribution of negative outcomes and START risk estimates (*n* = 181).

	**Negative outcomes**				**Specific risk estimates**							
	**Patient**		**Incident**		**Low**		**Moderate**		**High**		**Missing**	
	***n***	**%**	***n***	**%**	***n***	**%**	***n***	**%**	***n***	**%**	***n***	**%**
Self-harm	2	1.10	2	2.13	169	93.37	8	4.42	3	1.66	1	0.55
Suicide	7	3.87	9	9.57	159	87.85	17	9.39	3	1.66	2	1.10
Physical violence	6	3.31	19	20.2	155	85.64	23	12.71	3	1.66	2	1.10
Substance abuse	10	5.52	12	12.8	162	89.50	16	8.84	3	1.66	0	0
Victimization	2	1.10	2	2.13	166	91.71	15	8.29	0	0	0	0
Self-neglect	24	13.26	45	47.9	157	86.74	21	11.60	3	1.66	0	0
Unauthorized leave	4	2.21	5	5.32	171	94.48	10	5.52	0	0	0	0
Any START outcome	42	23.20	94	100								

### Predictive Validity of START

#### Presence of Negative Outcomes

Table 4 shows the predictive accuracy (AUC) of baseline START assessment scores for problematic behaviors in the 6 months after scoring. An AUC > 0.71 was considered as a large effect, 0.64~0.70 as medium, and 0.56~0.63 as small (26).

**Table 4:** Predictive accuracy (AUC) of baseline START assessment scores for problematic incidents in 6 months (*n* = 181).

	**Vulnerability total**		**Strength total**		**Specific risk estimate**	
	**AUC**	***p***	**AUC**	***p***	**AUC**	***p***
Self-harm	0.95^*^	0.029	0.64	0.51	0.98^*^	0.02
Suicide	0.83^**^	0.006	0.69	0.11	0.62	0.31
Physical violence	0.86^**^	0.001	0.82	0.007	0.79^*^	0.02
Substance abuse	0.78^**^	0.003	0.57	0.49	0.61	0.24
Victimization	0.72	0.28	0.75	0.22	0.46	0.84
Self-neglect	0.66^*^	0.012	0.61	0.094	0.69^**^	0.003
Unauthorized leave	0.78	0.056	0.83^*^	0.022	0.47	0.85
Any START outcome	0.74^***^	0.000	0.67^**^	0.001	N/A	N/A

*The AUC for Strength total predicts absence of negative outcomes*.

* *p < 0.05*,

** *p < 0.01*,

*** *p < 0.001*.

The vulnerability score significantly predicted occurrences of self-harm (AUC = 0.95, *p* = 0.03), suicide (AUC = 0.83, *p* < 0.01), physical violence (AUC = 0.85, *p* < 0.01), and substance abuse (AUC = 0.78, *p* < 0.01) with a large effect size. Feedback from participating RCs revealed the difficulty of assessing the intent to die for a given suicide/self-harm event. We therefore produced a composite self-harm/suicide outcome and the AUC by vulnerability score was 0.86 (*n* = 7, *p* < 0.01).

The Strength score significantly predicted only the non-occurrence of physical violence (AUC = 0.82, *p* < 0.01) and unauthorized leave (AUC = 0.82, *p* < 0.01).

SREs significantly predicted self-harm (AUC = 0.98, *p* < 0.05) and physical violence (AUC = 0.79, *p* < 0.01) with a large effect size, and self-neglect with a medium effect size (AUC = 0.69, *p* < 0.01), but not suicide, substance abuse, victimization, or unauthorized leave.

Both vulnerability score and strength score were predictive of “any START negative outcomes” with a medium to large effect size (AUC = 0.74, *p* < 0.01 for vulnerability score; AUC = 0.67, *p* < 0.01 for strength score).

#### Total Number and Types of Incidents per Patient

Vulnerability score correlated significantly with total number of incidents (Spearman's rho = 0.34, *p* < 0.01) and total types of incidents (Spearman's rho = 0.37, *p* < 0.01). Strength score also correlated significantly with the total number of incidents (Spearman's rho = −0.23, *p* < 0.01) and total types of incidents (Spearman's rho = −0.24, *p* < 0.01).

#### Internal Consistency

Cronbach's alpha for the standard 20 START items was 0.90 for vulnerability items and 0.91 for strength items.

## Discussion

This study appears to be the first to examine the validity of START in a prospective forensic sample living in the community and to explore the utility of START in Japan. Little research of this nature has been conducted outside North America and Europe.

### Predictive Validity

#### Physical Violence

START vulnerability score, strength score, and specific risk estimates all showed significant and high predictive validity for physical violence in the 6-month follow-up period. Past studies have consistently found that START was predictive of physical violence in 3–12 months (12, 27–32). START risk/vulnerability items for judging physical violence risk may thus also be generalizable to MDOs in Japan.

#### Self-Harm/Suicide

Vulnerability score showed predictive validity for both self-harm and suicide within 6 months, whereas strength score did not. O'Shea et al. (33) analyzed the predictive validity of START in an inpatient setting by combining self-harm and suicide, because their outcome data were derived from progress notes with a flag “self-harm/suicide” (33). This may reflect the difficulty in terms of clinical reality for distinguishing between deliberate self-harm with no intent to die and attempted suicide with intention to die (34). If this is true in inpatient settings, it is reasonable to assume that the difficulty would be larger in the community, where direct observation of patients' behaviors is much lower.

Our results found significant and sufficient AUCs in a 6-month follow-up period for the combined item of self-harm/suicide. Bearing in mind the significant and persistent risk of suicide following deliberate self-harm (35), relaxing the intention criteria may be more feasible in clinical settings, to judge combined risk estimates for self-harm/suicide. This is particularly true where the treated population consists primarily of individuals with psychosis, since these individuals are approximately six times more likely to die by suicide after a prior incident of deliberate self-harm (36).

#### Substance Abuse

Vulnerability scores, but not strength scores, were sufficiently predictive of substance abuse at 6 months. The vulnerability score outperformed the specific risk estimate for substance abuse. The distribution of risk estimates for substance use in this study was 162 patients with low risk (89.5%), 16 patients with medium risk (8.8%), and three patients with high risk (1.7%). However, actual incidents of substance abuse comprised nine cases (5%) in the 6-month follow-up period. This means that RCs tended to judge patients to be at a higher risk than they actual were (Fisher's exact test *p* = 0.004).

Past studies have documented that substance use tends to be a chronic condition where most patients need repeated treatment efforts (37). Therefore, when a patient is found to have a history of substance abuse, RCs might tend to consider and weigh this as evidence of elevated risk of further substance use. However, Japan has a low rate of drug use compared to other Western countries, such as the UK and European countries. For instance, according to a 2004 report from the World Health Organization, the 12-month prevalence of drug use disorders among male 15 years or older was 0.01% in Japan, markedly lower than the 1.29% in the UK and 1.14% in Canada (38). Our results suggest that the impact of historical substance use on future use may be mitigated in Japan because of the lower availability of drugs.

Making cultural adjustments when deciding on the impact of substance use may also be necessary for making clinical judgements regarding future violence in Japan. Past studies have repeatedly documented an effect of substance use on an elevated risk of violence in psychosis (39, 40). However, a recent study by Imai et al. (41) examined 420 Japanese patients with schizophrenia who had committed violent acts immediately prior to an emergency admission to a psychiatric hospital. Substance abuse and antisocial episodes were not recognized as significant violence-associated factors in that study. They speculated that this result was related to the markedly low rate of drug use in Japan (41). Taken together, evaluators in Japan should consider making cultural adjustments in weighing the impact of substance use when making clinical risk judgements. This is possible with START, which adopts an SPJ approach to risk assessment, where risks are estimated not by the total score, but by clinical judgements.

#### Unauthorized Leave

Inverted strength scores were predictive of future unauthorized leave, although vulnerability scores were not. This discrepancy could be attributed to the ambiguous definition of unauthorized leave in the community setting. For example, reported incidents have included temporary unauthorized leave (failing to report leaving) from a group home and unexplained disappearance for days where contact was impossible. Such instances of unauthorized leave may remain undetected in cases of independent living or when occurring between care coordination meetings.

#### Self-Neglect

Self-neglect was observed in 24 patients (13.3%), representing the most common START negative outcome in the study sample. Self-neglect was predicted by the vulnerability score and specific risk estimate, but not by the strength score. This was different from the observations of O'Shea et al. (12) who studied the predictive validity of START with inpatients and found neither vulnerability nor strength score predicted self-neglect. On the other hand, Marriott et al. reported different results that self-neglect in psychosis was predicted by both vulnerability score and strength score (42). As noted by Marriott et al. (42), the predictive validity of START for self-neglect may be influenced by the type of community setting. Our results may be reflective of Japanese MDOs residing in the community.

#### Victimization

Only two incidents (1.1%) of victimization were reported in our sample during the 6-month follow-up. Neither START vulnerability score nor strength score predicted their occurrence. These low rates can be interpreted as follows: The first is the underreporting of victimization. According to the International Crime Victims Survey by the United Nations Interregional Crime and Justice Research Institute in 2000, the yearly prevalence of victimization in 1999 was 15.2% in Japan (43). When also considering that victimization is higher for people with severe mental illness than for the general population (44, 45), the extent of underreporting in our sample is apparent. The second interpretation is that the compulsory nature of MTSA outpatient treatment may have served to protect against supervision. This aligns with a review of the effects of compulsory community treatment by Kisely et al. (46), who found that people receiving compulsory community treatment were less likely to be victims of violent or non-violent crime. They speculated that the effect may be due to the intensity of treatment or its compulsory nature (46).

#### Total Number and Types of START Outcomes

One of the assumptions of START is that risks overlap between negative outcomes (8). Vulnerability score correlated significantly with total number and types of START negative outcomes. Among the 42 patients who exhibited at least one START negative outcome within 6 months, 14 patients (34.14%) exhibited two or more types of START negative outcomes, supporting the assumption that risks overlap.

### Internal Consistency

Japanese versions of START items exhibited high internal consistency (>0.90), comparable to those in past Western studies (19, 27, 47).

### Vulnerability and Strength Scores

The fact that strength scores only showed moderately significant correlations with vulnerability scores suggests that START strengths do not merely represent the vulnerability/risk measure repeated and expressed in the opposite direction. This differed from the results described by Abidin et al. (27), where START vulnerability and strength scores were strongly and inversely correlated (*r* = −0.947) (27).

Strength score showed predictive validity only for physical violence and unauthorized leave. This was much less than that for the vulnerability score, which showed predictive validity for five of the seven outcomes. Two reasons may play roles in this difference. First, the vagueness of some START strength items may originate from the “lack of conceptual certitude around the relationship between protective and risk factors” (48). This reasoning may be supported by previous findings that assessment tools with separate items and unambiguous definitions for protective factors, such as SAPROF and SAVRY, tend to perform better in demonstrating incremental validity (49–51). Second, strength scores may be more predictive of positive results, such as job attainment and personal recovery, than merely non-negative results such as absence of violence. Our results may thus indicate the clinical utility of strength items as more relevant than risk estimates in guiding treatment planning.

### Limitations

This study shows several limitations that merit consideration when interpreting the results. First, inter-rater reliability was not determined in this study. All data were collected during the routine forensic probation practice of RCs, and it is not standard practice for MTSA patients to have two or more RCs in charge. Second, negative outcome data were collected from a single source, the RCs. Past studies have shown that detection of violence during follow-up increased steadily when combining methods (52). Our RCs obtained knowledge of forensic patients not only from direct contact with the patients in question, but also through care coordination meetings where multiple agencies and disciplines discuss the case. However, negative outcomes may still have been underreported. Aggression against psychiatric patients has been reported to show a tendency to be underreported (53), and the same conditions may have been present in the present study. This is important because the current study gathered outcome data for outpatients in the community, which is different from inpatient settings where outcome information is readily accessible and a strong obligation to record negative events is present. Future studies should ideally use collateral information on negative outcomes. Third, although the sample size of this study was the largest to date in validating risk assessment among forensic outpatients in Japan, the sample size was still too small to detect meaningful calculation of AUCs for victimization and unauthorized leave. Finally, although this study extended the evaluation of START to the outpatient population, the results remain limited to forensic psychiatric outpatients under MTSA in the community. The predictive validity of START in both forensic inpatients and general psychiatric patients in Japan remains unknown and is a target for future studies.

### Conclusion

The present study has major implications in terms of the dissemination of START in forensic psychiatric practice in the community. We were able to demonstrate via a prospective study design that START is an assessment tool that can be applied in Japan, a non-Western country. To conclude, this study advances our understanding regarding the utilization of START by clinicians in planning treatment for patients that will not only reduce the risks of negative outcomes, but also enhance strengths to promote recovery in the community.

## Data Availability Statement

The raw data supporting the conclusions of this article will be made available by the authors, without undue reservation.

## Ethics Statement

The study protocol was approved by the Ethics Committee of the National Center of Neurology and Psychiatry. All RCs provided written informed consent to participate in the research. Patients were notified of the study by posters in local probation offices and were guaranteed the right to opt out if they had any reservations about participating in the research. None of the participants opted out during the study period.

## Author Contributions

AK conceived and designed the present study. AK and MK collected data. AK, TK, and CF analyzed the data. AK drafted and revised the manuscript. AK and CF supervised the study. All authors approved the final manuscript.

## Conflict of Interest

AK receive royalties from texts or books she has published on risk assessment. The remaining authors declare that the research was conducted in the absence of any commercial or financial relationships that could be construed as a potential conflict of interest.
